# Supplementary material for: Chemical- and photo-activation of protein-protein thiol-ene coupling for protein profiling
Source: Commun Chem. 2025 Jan 29;8:25. doi: 10.1038/s42004-025-01412-6 (PMC11779957; doi:10.1038/s42004-025-01412-6)
Supplement: Supplementary file 2 — Supplemental Material [file 42004_2025_1412_MOESM2_ESM.pdf]

Supplementary Information

Chemical- and photo-activation of protein-protein thiol-ene coupling for protein profiling

André Campaniço[1], Marcin Baran[2], Andrew G. Bowie[2], Daniel B. Longley[3], Timothy Harrison[3], Joanna F. McGouran[1]*

[1]School of Chemistry, Trinity College Dublin, Trinity Biomedical Sciences Institute, 152-160 Pearse St., Dublin 2, Ireland
[2]School of Biochemistry and Immunology, Trinity College Dublin, Trinity Biomedical Sciences Institute, 152-160 Pearse St., Dublin 2, Ireland
[3]The Patrick G. Johnston Centre for Cancer Research, Queen's University Belfast, Belfast, UK

*Corresponding author
e-mail: jmcgoura@tcd.ie

Table of Contents

1. Supplementary Methods
2. Supplementary Figure S1. Initiation mechanism of the three initiators selected to trigger the thiol-ene coupling.
3. Supplementary Figure S2. Anti-HA Western Blot analysis of the incubation of the HA-$^{1\text{-}75}$Ub-alkene probe **1** with the recombinant DUB OTUB1, using different concentrations of $Mn(OAc)_3$ as the chemical initiator.
4. Supplementary Figure S3. Anti-HA Western Blot analysis of the incubation of the HA-$^{1\text{-}75}$Ub-alkene probe **1** with HEK293T cell lysates: role of degassing for initiators Irgacure 2959, Mes-Acr$^+$ and $Mn(OAc)_3$.
5. Supplementary Figure S4. Anti-HA Western Blot analysis of the incubation of the HA-$^{1\text{-}75}$Ub-alkene probe **1** with HEK293T cell lysates, using different concentrations of Mes-Acr$^+$ as the radical initiator.
6. Supplementary Figure S5. Anti-HA Western Blot analysis of the incubation of the HA-$^{1\text{-}75}$Ub-alkene probe **1** with USP7 and β-galactosidase.
7. Supplementary Figure S6. Uncropped Western Blots and SDS-PAGE, with protein ladder, for Figures 1-8 and S2-5.
8. Supplementary Figure S7. NMR spectra for compounds 4 and 5.
9. Supplementary references

1. Supplementary Methods

BCA Assay

The BCA assay was prepared in a 96-well plate. Standards of Bovine Serum Albumine (BSA) were prepared at the concentrations 2, 1.5, 1, 0.75, 0.5 and 0.25 mg/mL. 12.5 µL of each solution was applied to the 96-well plate in duplicate. 12.5 µL of each sample was also loaded into the plate in triplicate. A solution of BCA was prepared using BCA (BCA reagent A) and copper (II) sulphate (BCA reagent B) in a proportion on 50:1 and 100 µL of that solution was applied to each well. The plate was incubated at 37 °C with gentle shaking, for 30 min. The absorbance of each well was analysed at 562 nm, using the Nanodrop. The BSA concentration range was used to draw a calibration curve, that was used to calculate the concentration of each sample.

SDS-PAGE

Labelling samples were analysed on a 12% acrylamide gel (resolving gel: 1.3 mL 1.5 M Tris-Cl pH 6.8, 1.5 mL 40% acrylamide/bis-acrylamide (29:1), 2 mL dH_2O, 50 µL 10% SDS, 50 µL 10% ammonium persulfate (APS), 5 µL Tetramethylethylenediamine (TEMED); stacking gel: 630 µL 0.5 M Tris-Cl pH 6.8, 300 µL acrylamide/bis-acrylamide (29:1), 1.3 mL dH2O, 25 µL 10% SDS, 25 µL 10% APS, 2.5 µL TEMED). Fisher's EZ-RunTM Pre-Stained Rec Protein Ladder was loaded alongside the samples. Protein separation was performed at 150 V for 80 min in running buffer (25 mM Tris, 190 mM Gly, 1% SDS) and visualised by anti-HA western blotting.

Anti-HA Western Blotting

The transfer sandwich was prepared with a nitrocellulose membrane (GE Healthcare, Illinois USA) soaked in blotting transfer buffer (25 mM Tri, 190 mM Gly, 20% MeOH), filter papers and sponges. Proteins was performed in blotting transfer buffer for 18h at 15 V. Prior to immunoblotting, the membrane was incubated with blocking buffer (5% skimmed milk powder in PBS-Tween 20 0.1%) for 1 h at room temperature. The primary mouse monoclonal anti-HA antibody (1:2,000 dilution in blocking buffer) (Biolegend, California USA, Cat. Number 901501) was incubated with the membrane for 1 h, at room temperature, with gentle shaking. The membrane was then washed with PBS-Tween 20 0.1% (3 x 4 min) and PBS (2 x 4 min). The secondary antibody (1:4,000 dilution in blocking buffer) (Peroxidase conjugated AffiniPure Goat Anti-Mouse IgG (H+L), Jackson ImmunoResearch, Cambridgeshire UK, Cat. Number 115-035-166) was added to the membrane and incubated for 1 h at room temperature with gentle shaking. The membrane was then washed with PBS-Tween 20 0.1% (4 x 4 min), PBS (3 x 4 min) and dH2O (1 x 4 min). Chemiluminescence was visualised with Pierce ECL western blotting substrate (Thermofisher, Massachusetts USA). The membrane was imaged in a Chemidoc XRS+ (Biorad, California USA).

OTUB1 expression and purification

BL21 (DE3) cells transfected with a pET28a-LIC vector containing an N-terminal His6 tagged OTUB1 were cultured in LB medium (8 mL), containing kanamycin (100 µg/mL), for 18 h at 37 °C, 180 rpm. The resulting culture was transferred into fresh LB medium (300 mL), containing kanamycin (100 µg/mL), and grown at 37 °C at 180 rpm until an OD600 of 0.6 to 0.9 was reached. IPTG was then added at a final concentration of 0.4 mM and the bacteria were incubated for 20 h at 18 °C, 180 rpm. The cells were centrifuged at 6,000 rpm, for 15 min, and the resulting pellet was re-suspended in 25 mL of homogenate buffer (50 mM Tris-Cl pH 7.4, 5 mM $MgCl_2$, 250 mM sucrose) containing PMSF (20 µM). Cell lysis was performed using a sonication tip, for 5 min with a 3-sec pulse. The lysate was centrifuged at 13,000 rpm, for 45 min. Ni NTA agarose resin (Sigma-Aldrich, Missouri, USA) (1.5 mL, 1:1 suspension in 20 % EtOH) was centrifuged at 2,200 rpm for 5 min and the supernatant was discarded. dH_2O (2 x 0.7 mL) was added to the beads which were gently inverted until the resin was fully resuspended. The resulting solution was centrifuged at 2,200 rpm and the supernatant was discarded. Ni wash buffer (1.4 mL, 50 mM sodium phosphate pH 8.0, 300 mM NaCl, 10 mM imidazole) was added to the resin which was transferred to the clarified supernatant and incubated overnight at 4 °C with rolling. The resin was centrifuged at 2,200 rpm and the supernatant was discarded. Ni wash buffer (1 mL) was added to the resin which was fully resuspended by gentle inversion and centrifuged at 2,200 rpm for 5 min. This wash step was repeated four times. The supernatant was discarded after each washing step. Ni elution buffer A (4 x 0.7 mL; 50 mM sodium phosphate pH 8.0, 300 mM NaCl, 150 mM imidazole) was added to the resin that was resuspended by gentle inversion. The solution was centrifuged at 2,200 rpm for 5 min and the supernatants from these washes were pooled in clean microcentrifuge tubes. A final wash step was carried out with Ni elution buffer B (1 mL; 50 mM sodium phosphate pH 8.0, 300 mM NaCl, 300 mM imidazole) at 2,200 rpm for 5 min. The supernatant was discarded, and the beads were stored in 20% EtOH. The pooled supernatants were concentrated in a 10 kDa MW cut-off Vivaspin 500 centrifugal concentrators in a centrifuge at 9,000 rpm to a final concentration of 50 µL. Ni wash buffer (450 µL) was added to the tubes and concentrated to 50 µL in a centrifuge at 9,000 rpm. This wash step was repeated, and the solution was resuspended in storage buffer (150 µL, 20 mM Tris-Cl pH 8.0, 1mM DTT, 10% glycerol, 50 mM NaCl). The concentration was measured by nanodrop (12.81 µg/µL in 200 µL)[1,2].

HEK293T cell lysis

HEK293T (ECACC) cell lysis was performed using glass beads. Glass beads (100 µL) (0.5 mm, Sigma-Aldrich, Missouri, USA) and homogenate buffer (200 µL) were added to a HEK293T cell pellet (100 µL). Following resuspension, the mixture was vortexed in cycles of 20 s, followed by 90 s on ice. After 20 cycles, the cellular lysate and glass beads were centrifuged at 13,000 rpm for 5 min. The supernatant was collected and aliquoted. Final protein concentration was measured by Nanodrop (22.63 mg/mL, 200 µL).

2. Supplementary Figure S1. Initiation mechanism of the three initiators selected to trigger the thiol-ene coupling: a) Irgacure 2959[3]; b) 9-mesityl-10-methylacridinium perchlorate (Mes-Acr^{+})[4]; c) manganese (III) acetate[5].

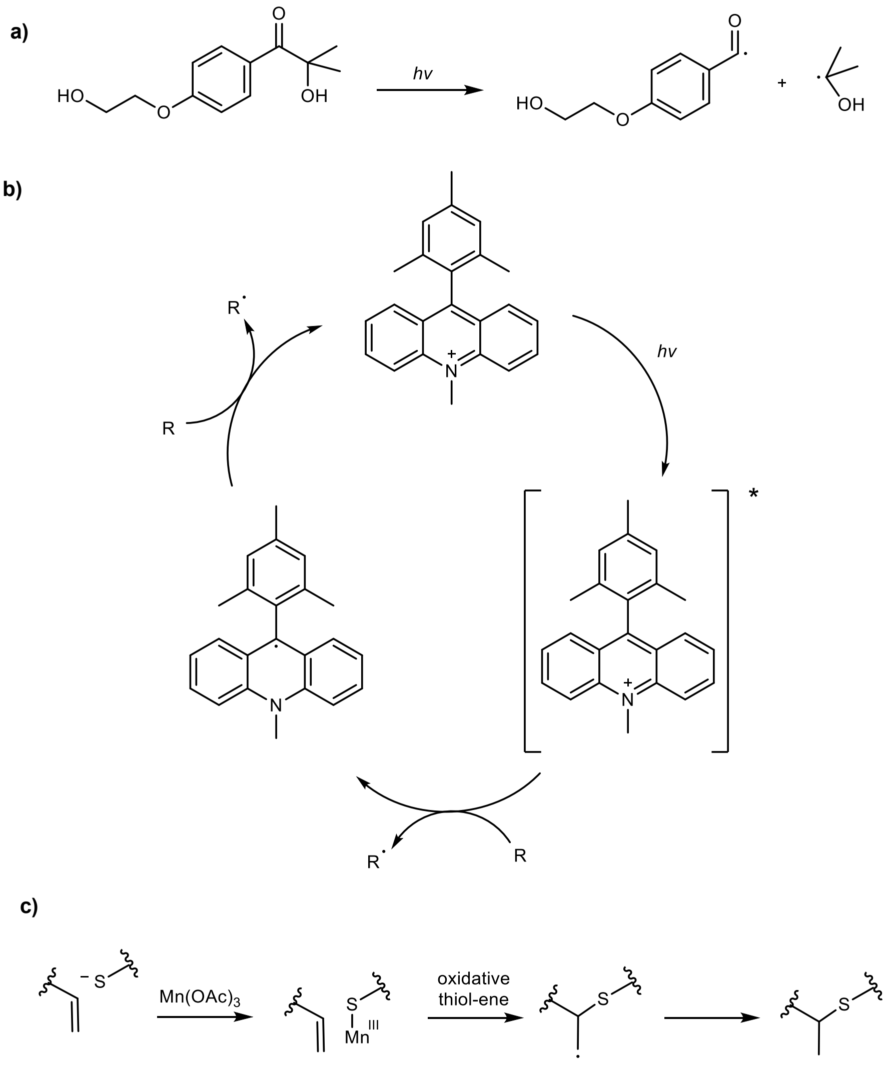

3. **Supplementary Figure S2. Anti-HA Western Blot analysis of the incubation of the HA-$^{1-75}$Ub-alkene probe 1 with the recombinant DUB OTUB1, using different concentrations of $Mn(OAc)_3$ as the chemical initiator.** HA-$^{1-75}$Ub-alkene probe **1** (2 ug) was incubated with the DUB OTUB1 (1 ug) for 90 minutes at 37 °C, prior to addition of $Mn(OAc)_3$ (2.5 µM - 250 µM) and a further 30 min incubation. A negative control without $Mn(OAc)_3$ and a positive control using a mixture of DPAP and MAP at a concentration of 500 µM were performed. The positive control was degassed and irradiated with UV, for 2 min. The results were analysed by SDS-PAGE and visualised by anti-HA Western Blot.

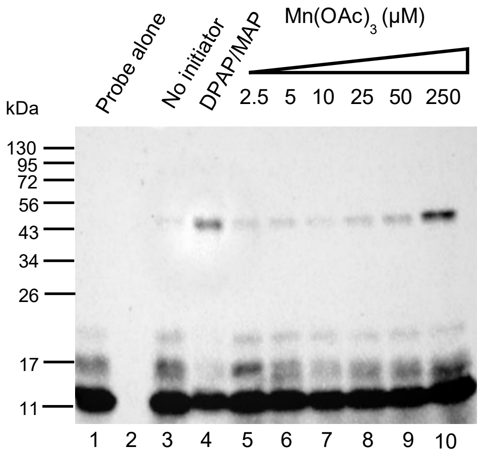

4. **Supplementary Figure S3. Anti-HA Western Blot analysis of the incubation of the HA-$^{1\text{-}75}$Ub-alkene probe 1 with HEK293T cell lysates: role of degassing for initiators Irgacure 2959, Mes-Acr^{+} and Mn(OAc)$_3$. .** HA-$^{1\text{-}75}$Ub-alkene probe **1** (2 µg) was incubated with HEK293T cell lysates (50 µg), for 90 minutes at 37 °C, prior to initiator addition. A negative control without initiator was performed. Role of degassing for initiators Irgacure 2959 (250 µM, 2-min UV irradiation), Mes-Acr^{+} (100 µM, 10-min blue light irradiation) and Mn(OAc)$_3$ (5 mM, 30-min incubation at 37 °C) was investigated. Non-degassed samples were used as the control and were compared with degassed samples. Results were analysed by SDS-PAGE and visualised by anti-HA Western Blot. Brightness was adjusted for lanes 8-9 with Mn(OAc)$_3$, to afford Figure 5.

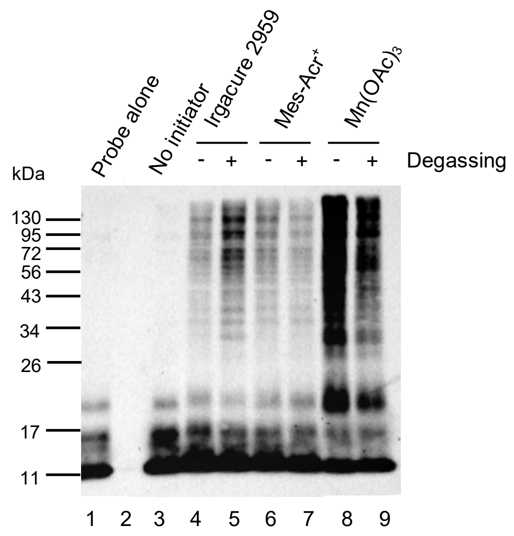

5. **Supplementary Figure S4. Anti-HA Western Blot analysis of the incubation of the HA-$^{1-75}$Ub-alkene probe 1 with HEK293T cell lysates, using different concentrations of Mes-Acr^{+} as the radical initiator.** HA-$^{1-75}$Ub-alkene probe **1** (2 µg) was incubated with HEK293T cell lysates (50 µg) for 90 minutes at 37 °C, prior to initiator addition. A negative control without initiator and a positive control using a mixture of DPAP and MAP at a concentration of 500 µM were performed. The positive control was degassed and irradiated with UV, for 2 min. A range of concentrations of Mes-Acr^{+} from 5 µM to 5 mM were tested. The samples were irradiated with a blue LED for 5 min, without previous degassing. Results were analysed by SDS-PAGE and visualised by anti-HA Western Blot.

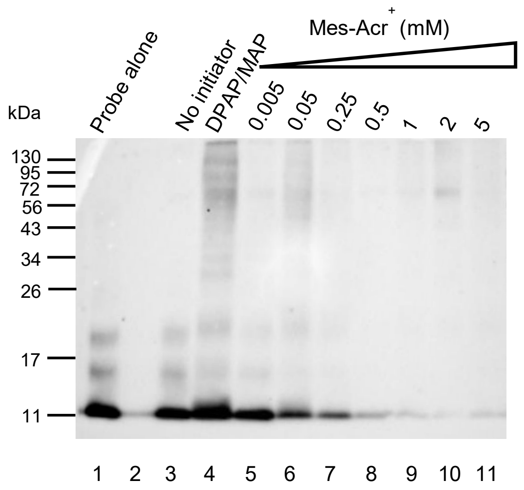

6. **Supplementary Figure S5. Anti-HA Western Blot analysis of the incubation of the HA-$^{1\text{-}75}$Ub-alkene probe 1 with USP7 and β-galactosidase.** HA-$^{1\text{-}75}$Ub-alkene probe 1 (2 µg) was incubated with the recombinant USP7 (4 µg) and β-galactosidase (5 µg), for 90 minutes at 37 °C, prior to the addition of Irgacure 2959 (250 µM, 2-min UV irradiation), Mes-Acr$^+$ (100 µM, 10-min blue light irradiation) or $Mn(OAc)_3$ (125 µM, 30-min incubation at 37 °C). A negative control without initiator was performed for each protein. Results were analysed by SDS-PAGE. a) Visualisation by anti-HA Western Blot, b) Visualisation by Silver Stain used as a control for the loading of both proteins (recombinant USP7 and β-galactosidase).

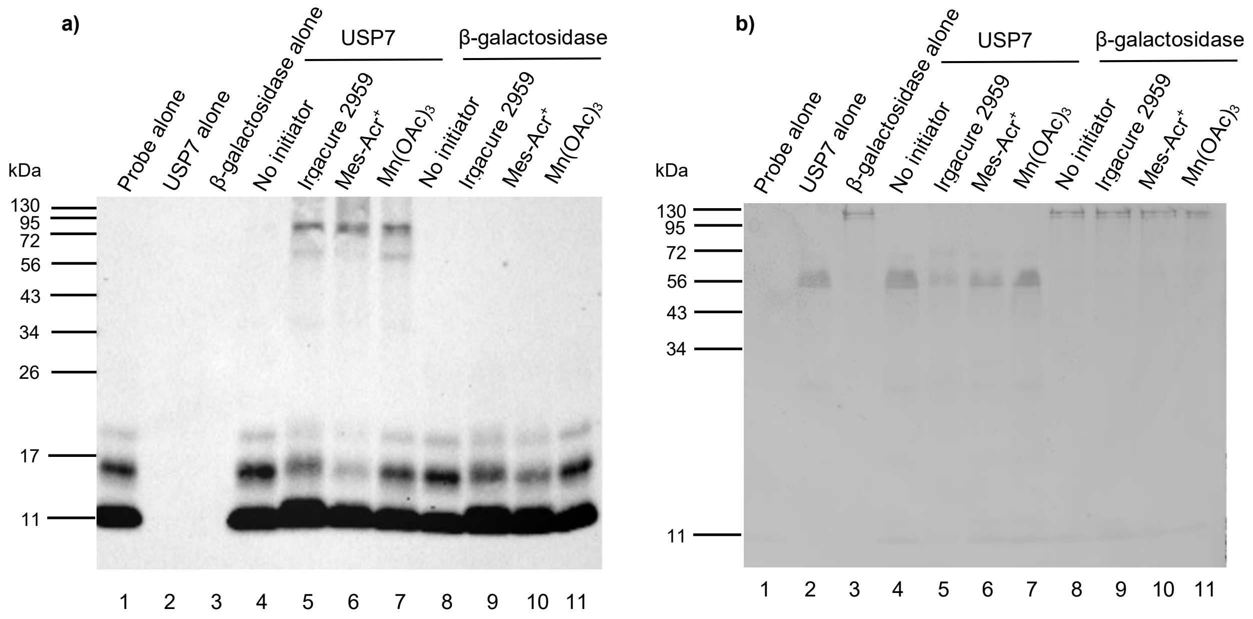

7. **Supplementary Figure S6. Uncropped Western Blots and SDS-PAGE, with protein ladder, for Figures 1-8 and S2-5.**

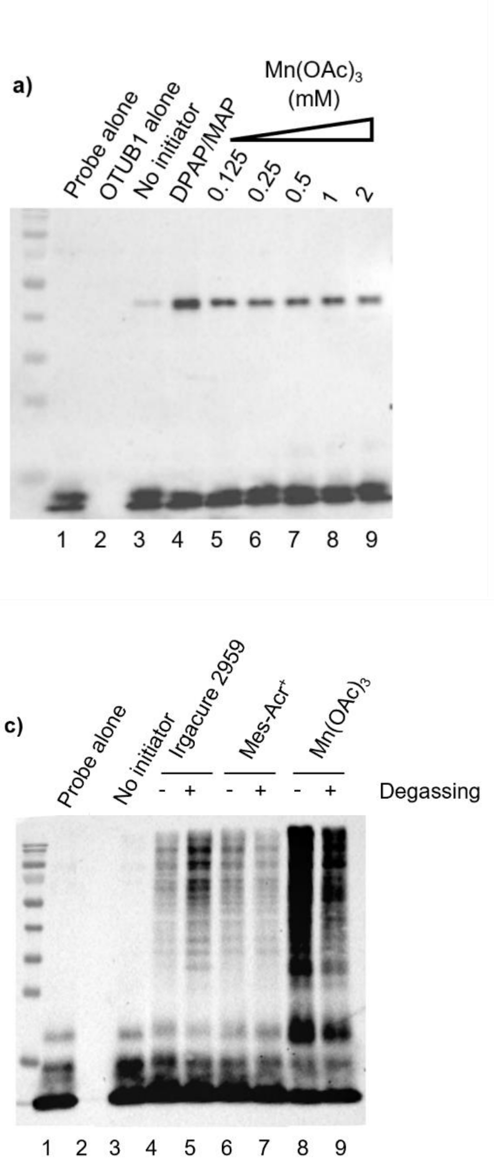

e)

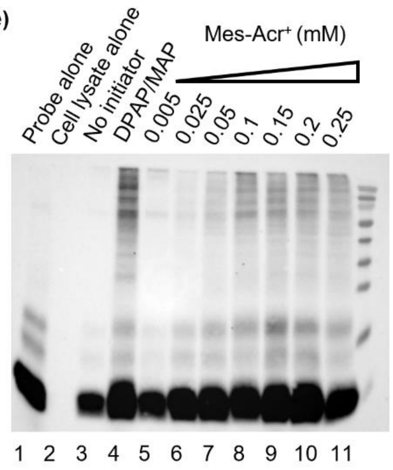
Mes-Acr⁺ (mM)
Probe alone
Cell lysate alone
No initiator
DPAP/MAP
0.005
0.025
0.05
0.1
0.15
0.2
0.25
1 2 3 4 5 6 7 8 9 10 11

f)

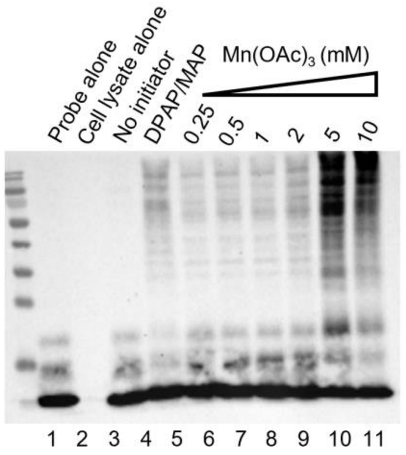
Mn(OAc)3 (mM)
Probe alone
Cell lysate alone
No initiator
DPAP/MAP
0.25
0.5
1
2
5
10
1 2 3 4 5 6 7 8 9 10 11

g)

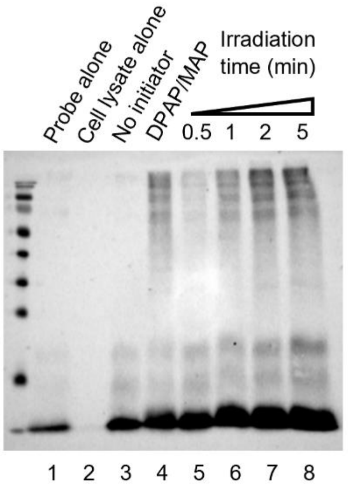
Probe alone
Cell lysate alone
No initiator
DPAP/MAP
Irradiation time (min)
0.5 1 2 5
1 2 3 4 5 6 7 8

h)

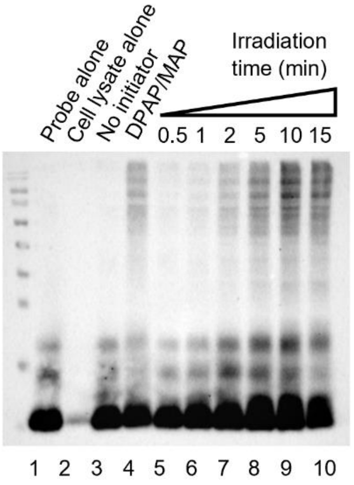
Probe alone
Cell lysate alone
No initiator
DPAP/MAP
Irradiation time (min)
0.5 1 2 5 10 15
1 2 3 4 5 6 7 8 9 10

i)

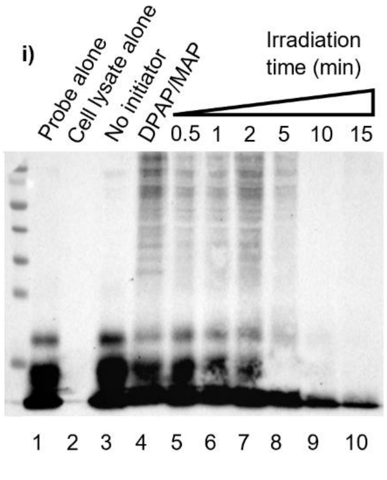
Probe alone
Cell lysate alone
No initiator
DPAP/MAP
Irradiation time (min)
0.5 1 2 5 10 15
1 2 3 4 5 6 7 8 9 10

j)

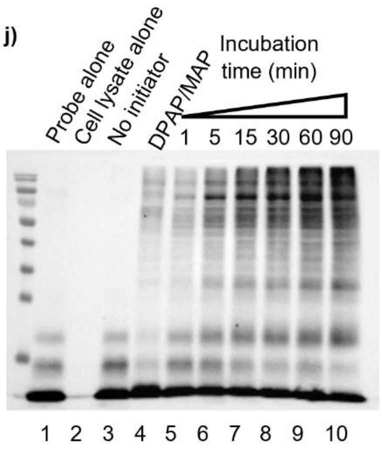
Probe alone
Cell lysate alone
No initiator
DPAP/MAP
Incubation time (min)
1 5 15 30 60 90
1 2 3 4 5 6 7 8 9 10

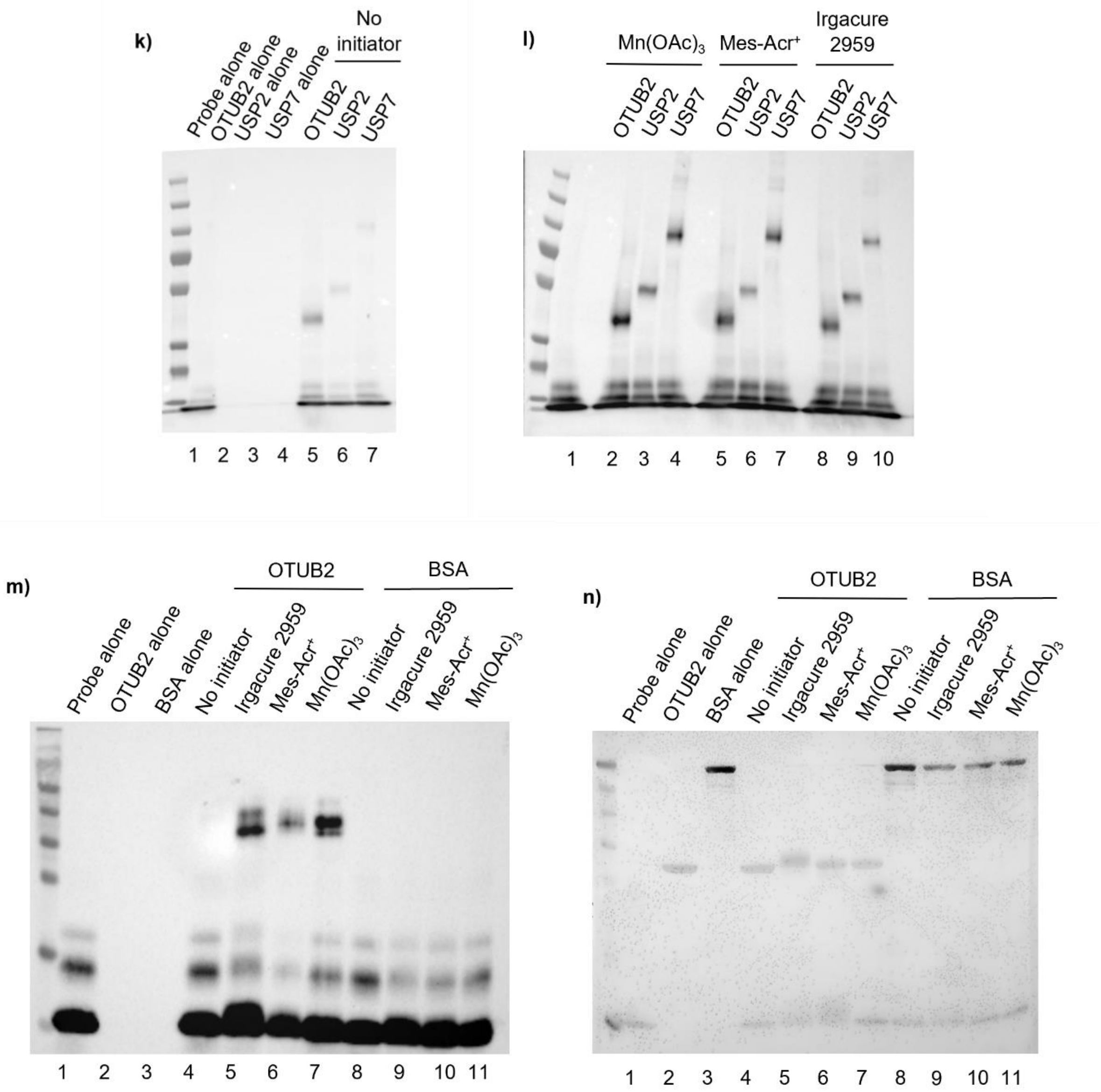
k)
Probe alone
OTUB2 alone
USP2 alone
USP7 alone
No initiator
OTUB2
USP2
USP7
1 2 3 4 5 6 7
l)
Mn(OAc)3
Mes-Acr+
Irgacure 2959
OTUB2
USP2
USP7
OTUB2
USP2
USP7
OTUB2
USP2
USP7
1 2 3 4 5 6 7 8 9 10
m)
OTUB2
BSA
Probe alone
OTUB2 alone
BSA alone
No initiator
Irgacure 2959
Mes-Acr+
Mn(OAc)3
No initiator
Irgacure 2959
Mes-Acr+
Mn(OAc)3
1 2 3 4 5 6 7 8 9 10 11
n)
OTUB2
BSA
Probe alone
OTUB2 alone
BSA alone
No initiator
Irgacure 2959
Mes-Acr+
Mn(OAc)3
No initiator
Irgacure 2959
Mes-Acr+
Mn(OAc)3
1 2 3 4 5 6 7 8 9 10 11

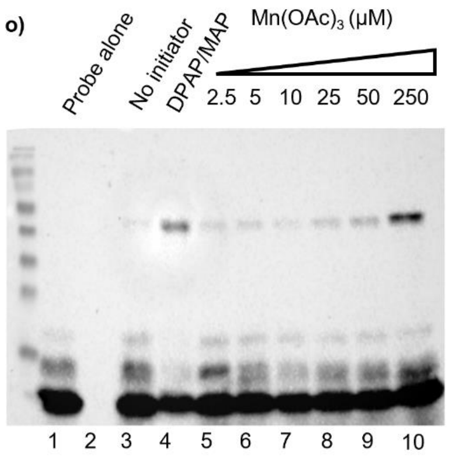
o)
Probe alone
No initiator
DPAP/MAP
Mn(OAc)3 (μM)
2.5 5 10 25 50 250
1 2 3 4 5 6 7 8 9 10

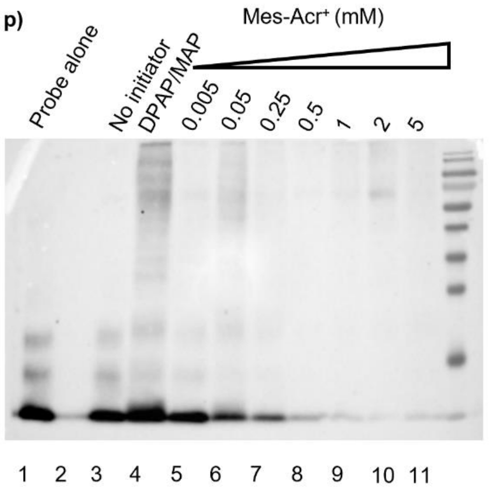
p)
Probe alone
No initiator
DPAP/MAP
Mes-Acr+ (mM)
0.005 0.05 0.25 0.5 1 2 5
1 2 3 4 5 6 7 8 9 10 11

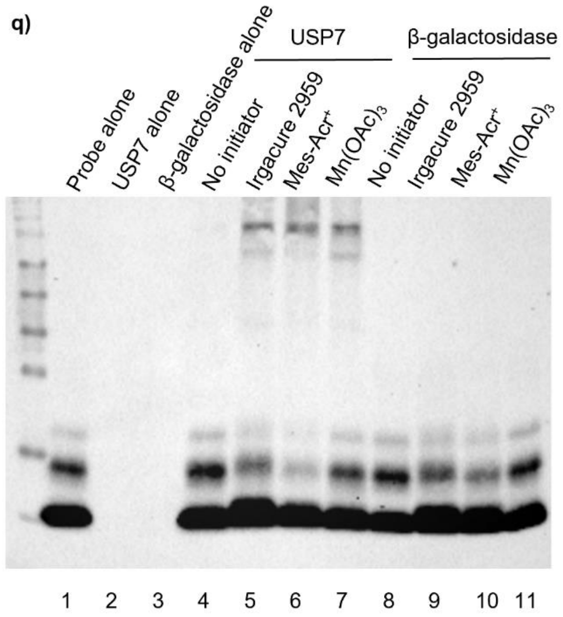
q)
USP7
β-galactosidase
Probe alone
USP7 alone
β-galactosidase alone
No initiator
Irgacure 2959
Mes-Acr+
Mn(OAc)3
No initiator
Irgacure 2959
Mes-Acr+
Mn(OAc)3
1 2 3 4 5 6 7 8 9 10 11

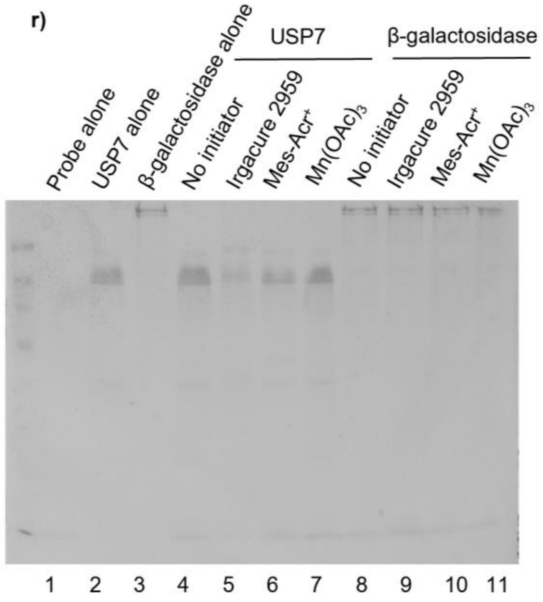
r)
USP7
β-galactosidase
Probe alone
USP7 alone
β-galactosidase alone
No initiator
Irgacure 2959
Mes-Acr+
Mn(OAc)3
No initiator
Irgacure 2959
Mes-Acr+
Mn(OAc)3
1 2 3 4 5 6 7 8 9 10 11

8. **Supplementary Figure S7. NMR spectra for compounds 4 and 5.** a) ^{1}H NMR for compound **4** (400 MHz, $CDCl_3$), b) ^{13}C NMR for compound **4** (125 MHz, $CDCl_3$), c) ^{1}H NMR for compound **5** (400 MHz, $CDCl_3$), d) ^{13}C NMR for compound **5** (125 MHz, $CDCl_3$).

a)

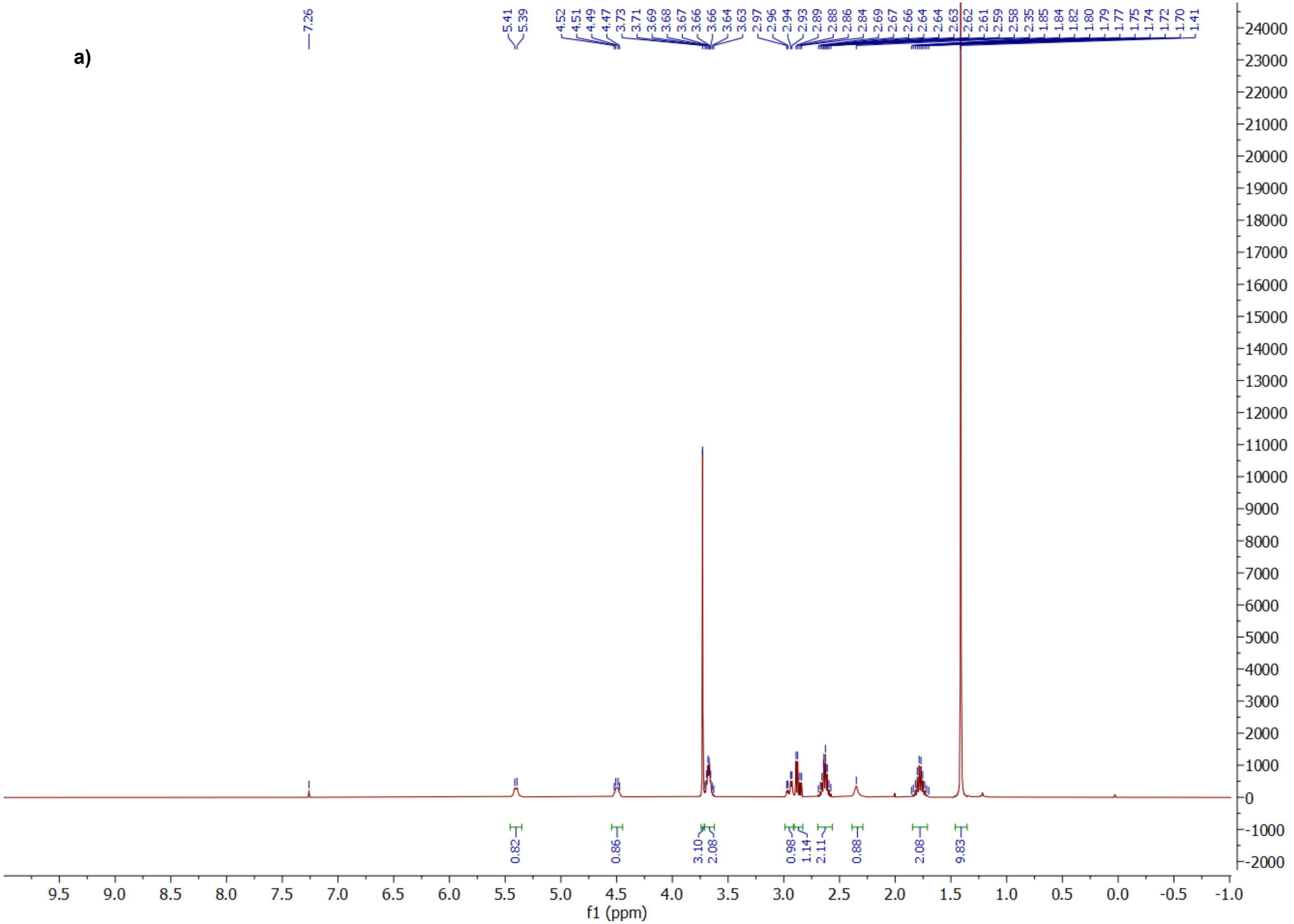

b)

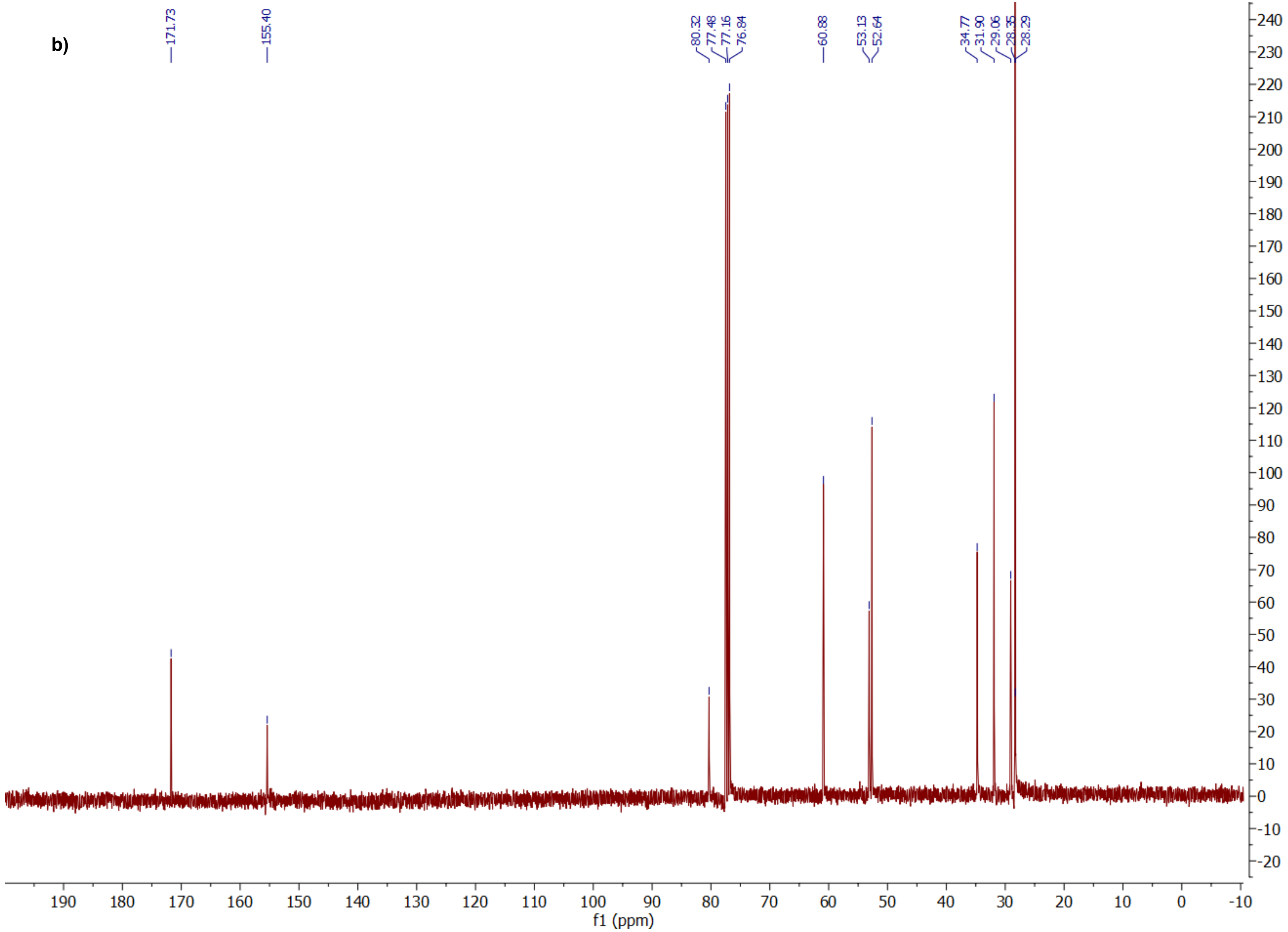

171.73
155.40
80.32
77.48
77.16
76.84
60.88
53.13
52.64
34.77
31.90
29.06
28.35
28.29
f1 (ppm)

c)

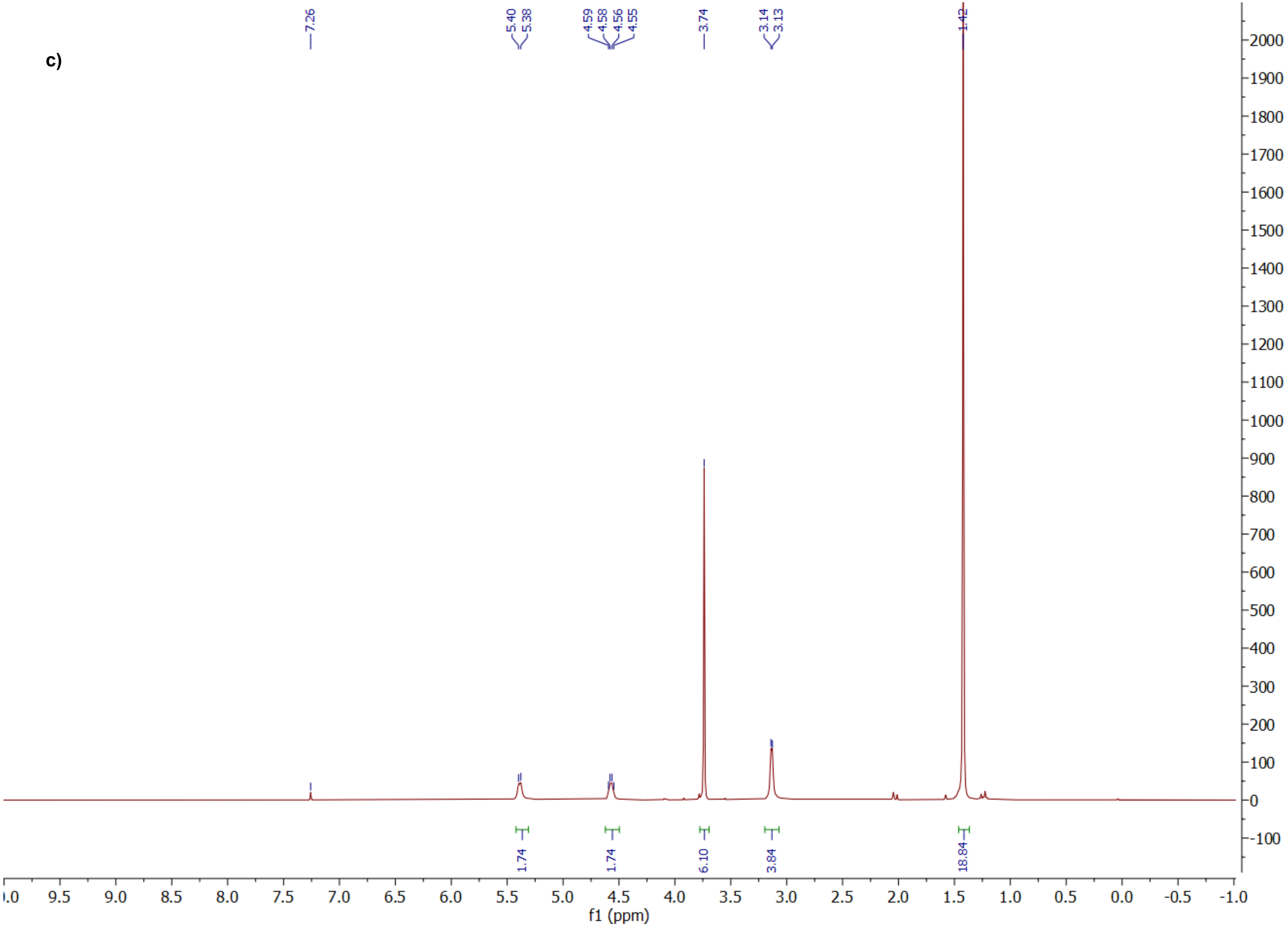
7.26
5.40
5.38
4.59
4.58
4.56
4.55
3.74
3.14
3.13
1.42
1.74
1.74
6.10
3.84
18.84
f1 (ppm)

d)

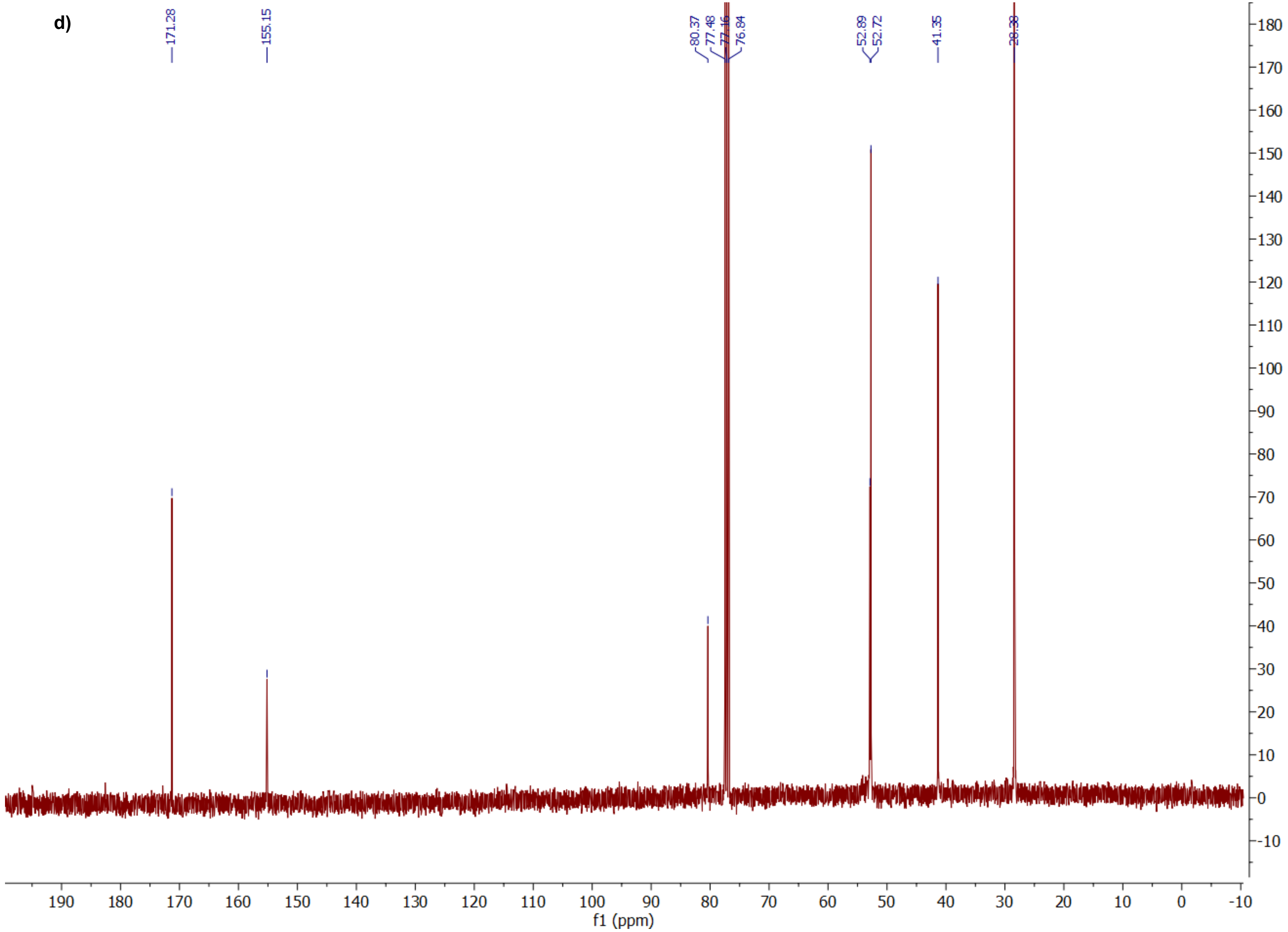

9. Supplementary References

1 Borodovsky, A. *et al.* Chemistry-based functional proteomics reveals novel members of the deubiquitinating enzyme family. *Chem Biol* **9**, 1149-1159 (2002). https://doi.org/10.1016/s1074-5521(02)00248-x

2 Chong, S. *et al.* Single-column purification of free recombinant proteins using a self-cleavable affinity tag derived from a protein splicing element. *Gene* **192**, 271-281 (1997). https://doi.org/10.1016/s0378-1119(97)00105-4

3 Tomal, W. & Ortyl, J. Water-Soluble Photoinitiators in Biomedical Applications. *Polymers (Basel)* **12** (2020). https://doi.org/10.3390/polym12051073

4 Romero, N. A. & Nicewicz, D. A. Mechanistic insight into the photoredox catalysis of anti-markovnikov alkene hydrofunctionalization reactions. *J Am Chem Soc* **136**, 17024-17035 (2014). https://doi.org/10.1021/ja506228u

5 Snider, B. B. Mechanisms of $Mn(OAc)_3$-based oxidative free-radical additions and cyclizations. *Tetrahedron* **65**, 10735-10744 (2009). https://doi.org/10.1016/j.tet.2009.09.025
